# Exploration of anti-oomycete components from wild plants against *Phytophthora infestans*

**DOI:** 10.3389/fpls.2026.1794332

**Published:** 2026-04-15

**Authors:** Saili Chang, He Bai, Anjin Qiao, Liangyan Liu

**Affiliations:** College of Agronomy and Biotechnology, Yunnan Agricultural University, Kunming, China

**Keywords:** anti-*Phytophthora infestans* activity, *Artemisia lavandulaefolia*, *Clinopodium repens*, LC-MS, plant extracts, potato late blight

## Abstract

**Introduction:**

Late blight caused by *Phytophthora infestans* severely threatens potato production, necessitating novel natural fungicide.

**Methods:**

Ethanol extracts from 11 wild plants were screened for anti-oomycete activity. Active extracts from *Artemisia lavandulaefolia* and *Clinopodium repens* were fractionated. The petroleum ether fraction and aqueous fraction were evaluated for effects on mycelial growth, morphology, spore germination, pathogenicity, stress response, and synergy with fungicide Infinito. Components were analyzed by LC-MS.

**Results:**

*A. lavandulaefolia* and *C. repens* extracts showed the highest inhibition (88.3% and 81.3%). the petroleum ether fraction of *A. lavandulaefolia* and the aqueous fraction of *C. repens* exhibited strong activity (IC_50_: 115.78 and 649.59 μg/mL), inhibited diverse isolates, and altered hyphal morphology. Spore germination reduced to 27.19% and 31.80%. Both fractions reduced tuber pathogenicity and showed stress tolerance. Combined with Infinito (IC_50_), enhanced inhibition was observed. LC-MS tentatively identified 20 components each, primarily flavonoids and phenolics.

**Discussion:**

This study elucidated that the petroleum ether fraction of *A. lavandulaefolia* and the aqueous fraction of *C. repens* are potential natural anti-oomycete resources, providing a basis for the development of environmentally friendly inhibitors against late blight.

## 1 Introduction

Potato (*Solanum tuberosum* L.) is an important dual-purpose crop for both staple food and vegetable, characterized by rich nutrition, wide adaptability, short growth cycle, and high comparative benefit. Its yield potential far exceeds that of traditional crops such as wheat, maize, and rice, making it a key economic crop for adjusting planting structure and enhancing agricultural efficiency in China (Shrestha et al., 2023; Rajendran et al., 2024). As the world’s largest potato producer, China cultivated 4.56 million hectares of potato in 2018, with a total production of 94.84 million metric tons, accounting for 49.9% of both global cultivation area and total potato output (Food and Agriculture Organization of the United Nations, 2024).

Late blight, caused by the oomycete *Phytophthora infestans* (Mont.) de Bary, remains the most devastating disease affecting potato yields. Under average conditions, it causes 10–20% yield losses, while severe outbreaks can reduce yields by 50–70% or even lead to total crop failure (Zhao et al., 2012). Potato late blight, first emerging as a devastating pandemic during the Irish famine of 1845, has now spread globally. It is widely regarded as the most destructive crop disease in the world, surpassing even rice blast and wheat rust in terms of its severity, challenges in control, and profound socio-economic impacts (Fry and Goodwin, 1997; Kamoun et al., 2015). Recognizing this threat, the International Potato Center (CIP) prioritized research on this pathogen, which culminated in the establishment of the Global Initiative for Late Blight (GILB) in 1996 to coordinate international efforts and mitigate crop losses (Forbes and Landeo, 2002). Since the 1990s, late blight outbreaks have become widespread and increasingly severe in China’s major potato-growing regions. In September 2020, the disease was classified as a “Category I crop pest” under China’s Regulations on Major Crop Pests and Diseases, underscoring its critical threat to production. Globally, it is estimated that annual yield losses and management costs attributable to late blight may reach up to US$ 3–10 billion (Dong and Zhou, 2022). Thus, developing safe and effective control measures for late blight is imperative for sustaining global potato production (Ministry of Agriculture and Rural Affairs of the People’s Republic of China, 2020).

Current strategies for controlling potato late blight primarily include rational cultivation practices, breeding for disease resistance, and chemical control. Rational cultivation practices involve scientific fertilization, crop rotation, intercropping, and staggered sowing to mitigate late blight damage. However, this approach is labor-intensive, requires extensive farming experience, and is constrained by geoclimatic conditions. Notably, it cannot eradicate *P.infestans*, the causal pathogen (Andrivon, 1994; Lamichhane et al., 2017). The methods of breeding for disease resistance utilize biotechnology, cell engineering, and genetic engineering to develop potato cultivars with high resistance to late blight. However, prolonged cultivation often leads to genetic degradation and declining disease resistance in these varieties (Fry, 2008). Chemical control, application of pesticides before or during outbreaks remains the most critical strategy. Commonly used formulations include mancozeb + metalaxyl (e.g., 72% metalaxyl-mancozeb wettable powder), fluazinam (e.g., 687.5 g/L fluopyram + propamocarb hydrochloride suspension), and cymoxanil (e.g., 50% cymoxanil wettable powder) (Wang X. G. et al., 2025; Liu et al., 2024; Zhu et al., 2024). While effective, these synthetic pesticides exhibit high toxicity, slow environmental degradation, and pose significant risks to human health and ecosystems with long-term use. In response to growing concerns about food safety and environmental sustainability, the development of biopesticides pesticides—characterized by low toxicity, rapid biodegradability, and high selectivity—has emerged as a critical direction for modern agricultural innovation (Pimentel, 2005; Dayan et al., 2009).

Yunnan boasts rich plant resources, with 19,333 species of higher plants, accounting for 50.1% of the national total, including 8,772 endemic species in China. The wild plant richness of Yunnan ranks first in the country, constituting a critical strategic resource for China (Yunnan Provincial People's Government Portal, 2025). During long-term environmental adaptation and evolutionary processes, plants have developed safe, efficient, and structurally diverse defensive components to combat pathogenic infections, serving as an important source for the discovery of novel antimicrobial agents (Hua, 2024; Isah, 2019; Luo et al., 2024). Gupta et al.(2002) found that leaf extracts of Eupatorium ayapana (Asteraceae) exhibited antimicrobial activity against Bacillus subtilis and Alternaria solani. Yanar et al. (2011) reported that extracts from Xanthium strumarium, Lauris nobilis, Salvia officinalis and Styrax officinalis significantly inhibited the mycelial growth of *P. infestans*. Rogozhin et al. (2020) discovered that biomass extracts from Equisetum arvense exhibit inhibitory activity against *P. infestans*. Blaeser and Steiner (1999) reported that Potentilla eracta and Salvia officinalis extracts exhibited high anti-oomycete activities against *P. infestans* on tomato plants under greenhouse conditions. Krebs and Forrer (2001) found that extracts from Malvae folium, Salviae folium (S. officinalis) and Bardanae radix (Arctium lappa) roots significantly reduced the incidence of late blight. Muto et al. (2005) tested the extracts derived from fresh and dry tissues of 14 plant species against *P. infestans* and Alternaria solani. Baka (2014) identified anti-oomycete activity against tomato late blight in extracts from five wild medicinal plants in Egypt.

This study aims to conduct a preliminary screening of the anti-oomycete activity of ethanol extracts from 11 wild plant species against *P. infestans*, in order to select plant materials exhibiting significant inhibitory effects on mycelial growth. Subsequently, the extracts with superior activity will be subjected to gradient liquid–liquid partitioning using petroleum ether, chloroform, and ethyl acetate to determine the most effective polarity fraction for inhibiting the pathogen. This phase of research will systematically evaluate the effects of the active fraction on mycelial growth, hyphal morphology, spore germination capacity, pathogenicity, stress response analysis, and combined treatment experiments with fungicde, infinito. Building on these findings, the optimal anti-oomycete polarity fraction will be subjected to chemical composition analysis via liquid chromatography–mass spectrometry (LC–MS) to preliminarily identify potential bioactive components against *P. infestans*. The study is expected to provide a theoretical foundation for the development of efficient, low–toxicity, botanically–based agents for the control of potato late blight.

## 2 Materials and methods

### 2.1 Plant materials

Plant materials were collected in March 2023 from Changchong Mountain, Kunming City, Yunnan Province, China (24°23′–26°33′ N, 102°10′–103°40′ E). Taxonomic identification was performed by Researcher Geng Chang’an from the Kunming Institute of Botany, Chinese Academy of Sciences, based on morphological characteristics. The scientific names were verified against the *Flora of China* (http://www.iplant.cn/foc) and *Plants of the World Online* (https://powo.science.kew.org). Voucher specimens are deposited at the Key Laboratory of Crop Germplasm Innovation and Sustainable Utilization of Higher Education Institutions in Yunnan Province, Yunnan Agricultural University. Detailed information on the plant species is provided in **Table 1**. List of wild plant species collected for anti-oomycete screening against *Phytophthora infestans* The potato cultivar used in this study was ‘Qingshu 9’, for which healthy and robust tubers were selected.

**Table 1:** List of wild plant species collected for anti-oomycete screening against *Phytophthora infestans*.

Scientific name	Common name	Family	Plant part used	Voucher specim no.
*Artemisia lavandulaefolia* DC.	Lavender-leaved Wormwood	Asteraceae	Aerial parts	PLB-2023-03001
*Clinopodium repens* (Roxb.) Wall. ex Benth.	Creeping Savory	Lamiaceae	Aerial parts	PLB-2023-03002
*Senecio scandens* Buch.-Ham. ex D.Don	Climbing Groundsel	Asteraceae	Aerial parts	PLB-2023-03003
*Debregeasia orientalis* C.J.Chen	Oriental Debregeasia	Urticaceae	Aerial parts	PLB-2023-03004
*Galinsoga quadriradiata* Ruiz & Pav.	Shaggy Soldier	Asteraceae	Aerial parts	PLB-2023-03005
*Aster baccharoides* (Benth.) Steetz.	Baccharis-like Aster	Asteraceae	Aerial parts	PLB-2023-03006
*Sonchus oleraceus* L.	Common Sowthistle	Asteraceae	Aerial parts	PLB-2023-03007
*Ageratina adenophora* (Spreng.) R.M.King & H.Rob.	Crofton Weed	Asteraceae	Aerial parts	PLB-2023-03008
*Synedrella nodiflora* (L.) Gaertn.	Nodeweed	Asteraceae	Aerial parts	PLB-2023-03009
*Laggera crispata* (Vahl) Hepper & J.R.I. Wood	Crisped Laggera	Asteraceae	Aerial parts	PLB-2023-030010
*Eschenbachia japonica* (Thunb.) J.Kost.	Horseweed	Asteraceae	Aerial parts	PLB-2023-030011

### 2.2 Tested strain and culture conditions

The *Phytophthora infestans* isolates, including T30-4 (A2 mating type) obtained from the Shanghai Biological Technology Center (SHBCC), 88069 (self-fertile) purchased from Hangzhou Fengxun Biotechnology Co., Ltd., SW98-2 (A1 mating type) provided by the Late Blight Research Laboratory of Hebei Agricultural University, and P12103 (self-fertile) supplied by Professor Shengbiao Hu of Hunan Normal University, were cultured on rye medium at 18–20 °C for 10–15 days (Rani et al., 2023).

### 2.3 Tested fungicide

The tested fungicide was Infinito, with the active ingredients fluopicolide and propamocarb hydrochl-oride. It is manufactured by Bayer CropScience, with the country of origin being India.

### 2.4 Processing of plant materials and preparation of extracts

The aerial parts of the plants were washed thoroughly and air-dried under natural shade conditions. After drying, they were pulverized using a plant grinder, and the resulting powder was stored in sealed bags for subsequent use. Ethanol extracts were prepared by cold maceration (Jiang et al., 2018). Specifically, 2 kg of plant powder was placed in an Erlenmeyer flask and soaked with 10 volumes of 85% ethanol at room temperature for 48 h, with manual stirring three times daily. The extraction was repeated twice, and the combined filtrate was concentrated under reduced pressure at 45 °C using a rotary evaporator (OSB-2100, Shanghai Ailang Instruments Co., Ltd, China) to obtain the plant extract.

### 2.5 Screening of anti-oomycete activity of plant extracts

Plant extracts were dissolved in a mixture of 0.5 mL dimethyl sulfoxide (DMSO) and 0.5 mL sterile distilled water to prepare rye agar plates with a final extract concentration of 1 mg/mL as the treatment group. Control plates (CK) without extract and negative control plates containing the same concentration of DMSO were also prepared. A 6-mm mycelial disk of *P. infestans* strain T30–4 was placed at the center of each plate. After 4 days of growth, the colony diameter was measured daily for 6 consecutive days using the two perpendicular diameters method. The formula for calculating the inhibition rate is as follows, Three replicates were set for each concentration (Zhu et al., 2023). The plant extract with the highest anti-oomycete activity was selected. The selected extract was then prepared into rye agar plates with concentration gradients of 0.5, 1, and 2 mg/mL using the method described above, and its inhibition rate was determined with three biological replicates per concentration.


Inhibition Rate (%)=(C−T/C−0.6)×100

Where C represents the mycelial colony diameter observed on the control plate, T represents the colony diameter observed on the medium supplemented with the extract, and 0.6 denotes the diameter (cm) of the mycelial plug.

### 2.6 Gradient extraction of the best anti-oomycete plant extract and antimicrobial assay of different polarity fractions

The plant extracts exhibiting superior anti-oomycete activity (30 g) were suspended in a 10-fold volume of sterile water (300 mL). This suspension was then sequentially subjected to gradient extraction with petroleum ether, chloroform, and ethyl acetate. This process yielded four fractions of distinct polarities: the petroleum ether fraction (PE), chloroform fraction (CHCl_3_), ethyl acetate fraction (EA), and the remaining aqueous fraction (AQ). All fractions were concentrated to a paste under reduced pressure at 45 °C and stored at -4 °C for subsequent use (Wang et al., 2007). Rye agar plates containing the different polarity fractions at a concentration of 1 mg/mL were prepared as treatments. The anti-oomycete activity against *P. infestans* strain T30–4 was determined using the method described in section 2.5, with three biological replicates performed for each treatment, to screen for the most effective anti-oomycete polarity fraction.

### 2.7 Determination of the half maximal inhibitory concentration (IC_50_) of the best anti-oomycete polarity fraction

Based on the results from section 2.6, the optimal anti-oomycete polar fraction were prepared at concentrations of 2000, 1000, 500, 250, 125, and 62.5 μg/mL in rye agar medium. The above procedure was repeated to calculate the inhibition rate against T30-4. The IC_50_ value of the extract against T30–4 was determined using SPSS, version 25 (IBM Corp., Armonk, NY, USA) (Zhu et al., 2023). Subsequently, the IC_50_ concentration of the extract against T30–4 was used to assess its anti-oomycete activity against the isolate 88069, P12103, SW98-2, with three biological replicates performed.

### 2.8 Effects of the optimal anti-oomycete polar fraction on mycelial morphology

The effect of the optimal anti-oomycete polar fraction on the mycelial morphology of *P. infestans* strain T30–4 was investigated using media prepared at a final concentration corresponding to the IC_50_ value. T30–4 was inoculated onto these media and inoculated for 7 days. Subsequently, samples were prepared for scanning electron microscopy (SEM) observation. Mycelia from different treatments were fixed with 2.5% glutaraldehyde at 4 °C for 2 h, followed by three washes (15 min each) with 0.1 M phosphate buffer (pH 7.2). After fixation, the samples were dehydrated through a graded ethanol series (30%, 50%, 70%, 80%, 90%, and twice in 100%) for 15 minutes at each step. They were then sequentially washed for 10 minutes each with mixtures of ethanol and tert-butyl alcohol at ratios of 3:1, 1:1, and 1:3, followed by two 10-minute immersions in pure tert-butyl alcohol. The samples were freeze-dried using a freeze dryer (Model ES-2030, Hitachi, Japan) and coated with gold for 60 seconds using a sputter coater (E-1010, Hitachi, Japan). All samples were observed under a scanning electron microscope (Model SU8100, Hitachi, Japan) at an accelerating voltage of 3 kV and a magnification of 6000× (Soylu et al., 2006).

### 2.9 Effect of the optimal anti-oomycete polar fraction on sporangial germination

Ten-day-old cultures of *P. infestans* strain T30–4 were flooded with sterile water and allowed to stand for 10 min. The resulting suspension was filtered through a 15 μm nylon mesh to collect sporangia, and the collected sporangia were diluted to prepare a sporangial suspension at a concentration of 1 × 10^6^ sporangia/mL.In a 96-well plate, 20 μL of sporangia suspension was added to each well and treated with 80 μL of extract at concentrations of 1/2 IC_50_ and IC_50_, respectively, while an equal volume of sterile water was added as a CK. After incubation for 12 h, sporangial germination was observed under a microscope. Each treatment was performed with three biological replicates (Zhu et al., 2023). The sporangial germination rate was calculated using the following formula:


  sporangial Germination Rate (%)=(G/T)×100

Here, G represents the number of germinated sporangia, and T represents the total number of sporangia observed.

### 2.10 Effect of the optimal anti-oomycete polar fraction on the pathogenicity of *P. infestans* T30–4 on potato tubers

Potato tubers(cultivar ‘Qingshu 9’) were cut into pieces measuring 4×3×0.6 cm. These pieces were surface-disinfested by immersion in 75% ethanol for 2 min, followed by rinsing 3–4 times with sterile distilled water, and then air-dried in a laminar flow cabinet. A single wound (4 mm in diameter) was made at the center of each tuber piece using a sterile cork borer. Mycelial plugs of *P. infestans* isolate T30-4, cultured for 10 days as described in section 2.7, were placed into the wound of each tuber piece. Three biological replicates were included for each treatment. The inoculated tuber pieces were placed in Petri dishes lined with moistened filter paper and incubated in darkness at 20 °C for 5 days. Following incubation, the tubers were photographed, and the proportion of the lesion size relative to the total tuber piece area was calculated using ImageJ software (NIH, Bethesda, MD, USA) (Zhu et al., 2023).

### 2.11 Stress analysis of *P. infestans* T30–4 treated with the optimal anti-oomycete polar fraction

*P. infestans* T30–4 was subjected to various stress conditions: NaCl (0.1 mol/L) for 10 min, H_2_O_2_ (0.2 mL/L) for 10 min, UV irradiation (1350 µW mm^-^²) for 10 min, cold stress at 4 °C for 24 h, and heat stress at 37 °C for 2 h. Following each treatment, the mycelial plugs were inoculated onto rye agar plates without any extract (control) and onto rye agar plates containing the optimal anti-oomycete polar fraction at its IC_50_ concentration. All plates were incubated for 10 days. The method for determining the inhibition rate and the calculation formula were the same as those described in section 2.5. All treatments were performed with three biological replicates (Zhu et al., 2023).

### 2.12 Combined assay of the optimal anti-oomycete polar fraction and fungicide

Rye agar plates were prepared with the following treatments: CK, the optimal anti-oomycete polar fraction (IC_50_), Infinito (3 µL/L), Infinito (6 µL/L), Infinito (9 µL/L), Infinito combined with the optimal anti-oomycete polar fraction (3 µL/L + IC_50_), Infinito combined with the optimal anti-oomycete polar fraction (6 µL/L + IC_50_), and Infinito combined with the optimal anti-oomycete polar fraction (9 µL/L + IC_50_). After 10 days of incubation, the colony diameter was measured, and the inhibition rate was calculated using the same method described previously (Zhu et al., 2023). The formula established by Jin (2004) was employed to assess the combined effects of the two agents, as follows:


Q=E(A+B)/(EA+EB−EA×EB)

where EA, EB, and E(A+B) represent the inhibition rates of agent A alone, agent B alone, and their combination, respectively. A Q value < 0.85 indicates antagonism, 0.85–1.15 indicates an additive effect, and ≥ 1.15 indicates synergism. All treatments were performed with three replicates.

### 2.13 LC-MS analysis of the plant active fraction

The polar fraction exhibiting the most significant anti-oomycete activity was selected for LC-MS analysis. It was fully dissolved in methanol (chromatographic grade), filtered through a 0.22 µm microporous membrane, transferred to a sample vial, and analyzed using a liquid chromatography-mass spectrometry system (Agilent 1290 UPLC/6540 Q-TOF).

Chromatographic conditions: ZORBAX SB-C18 column (4.6 mm × 250 mm, 5 µm); mobile phase consisting of acetonitrile (A) and 0.1% formic acid aqueous solution (B); gradient elution: 0–1 min, 5% A; 1–8 min, 5%–24% A; 8–16 min, 24%–48% A; 16–25 min, 48%–78% A; 25–30 min, 78%–100% A. The column temperature was maintained at 30 °C, the flow rate was 0.3 mL/min, and the injection volume was 1 µL.

Mass spectrometric conditions: Electrospray ionization (ESI) source in positive and negative ion MSE scanning mode; cone voltage: 40 V; source offset: 80 V; ion source temperature: 100 °C; desolvation gas temperature: 500 °C; cone gas flow (N2): 50 L/h; desolvation gas flow (N2): 800 L/h; sampling cone voltage: 40 V; low collision energy: 6 V; high collision energy ramping from 40 to 60 V.

### 2.14 Statistical analysis

Experimental data were processed and preliminarily analyzed using Excel 2019, followed by one-way ANOVA using SPSS, version 25 (IBM Corp.). Tukey’s honest significant difference test was employed to assess the significance of differences among datasets. Finally, graphs and charts were generated using Origin 2024 to visually represent the results (Liu et al., 2022).

## 3 Results

### 3.1 Preliminary screening of anti-oomycete activity of extracts from 11 wild plant species against T30-4

**Figure 1:**
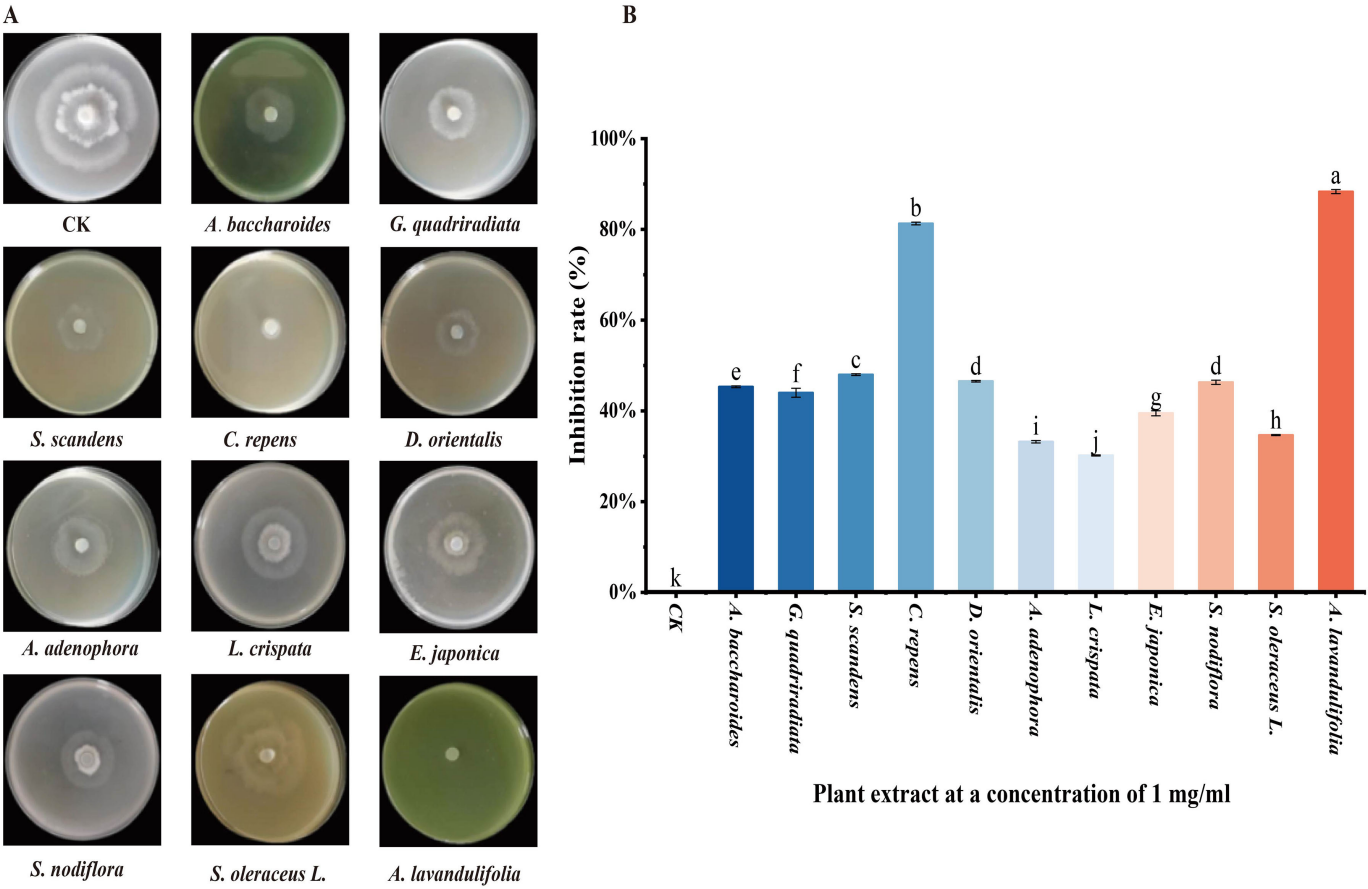
Inhibition of mycelial growth of T30–4 by 11 wild plant extracts at a concentration of 1 mg/ml. **(A)** Mycelial growth of T30–4 treated with 11 plant extracts. **(B)** Inhibition rates of 11 wild plant extracts on mycelial growth of T30-4. Different lowercase letters indicate significant differences (P < 0.05). P-values were calculated using one-way ANOVA. Each value represents the mean ± SD.

**Figure 2:**
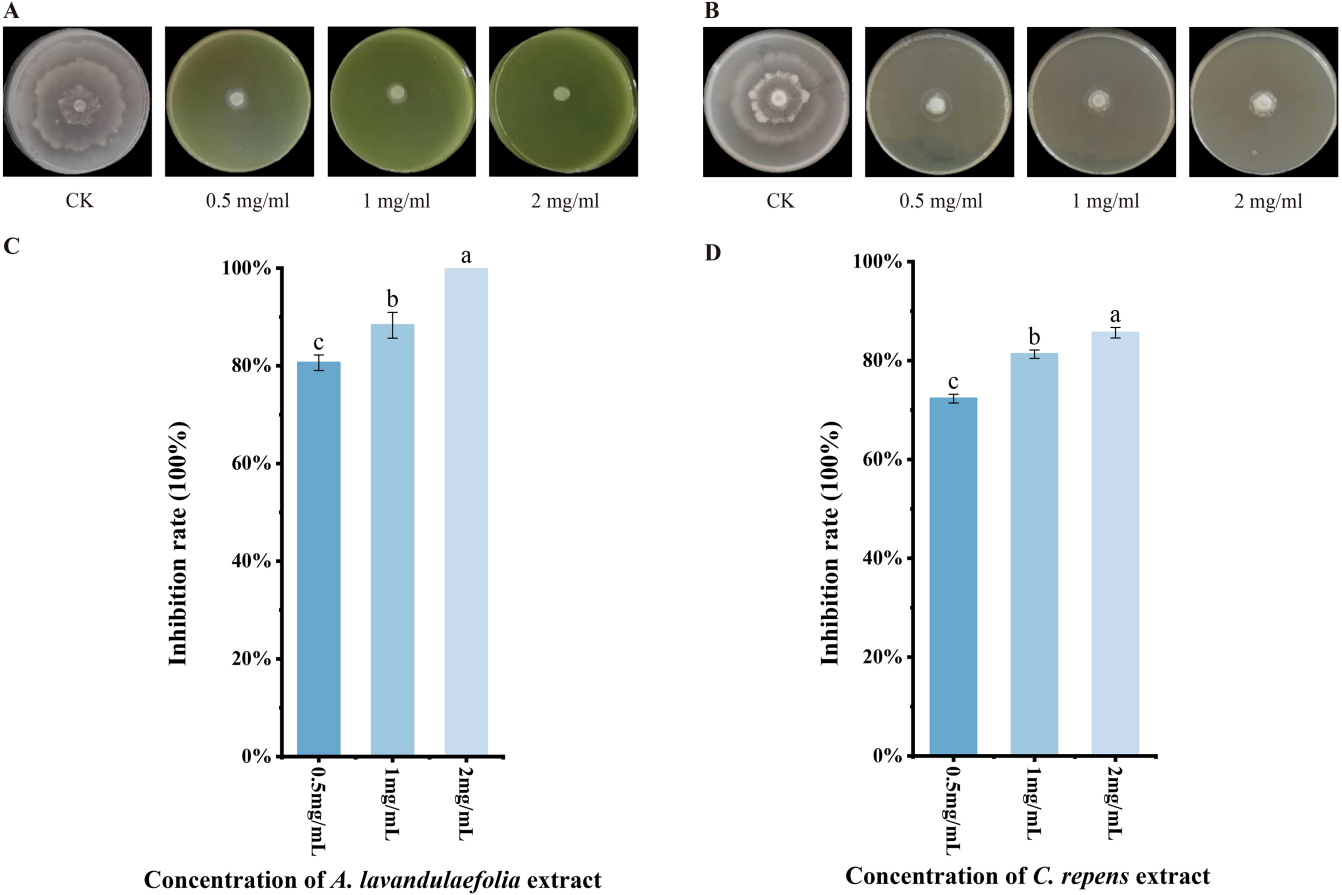
Inhibition of T30–4 mycelial growth by extracts from *A. lavandulaefolia* and *C. repens* at different concentrations. **(A)** Mycelial growth of T30–4 treated with different concentrations of *A. lavandulaefolia* extract. **(B)** Mycelial growth of T30–4 treated with different concentrations of *C. repens* extract. **(C)** Inhibition rate of *A. lavandulaefolia* extract on T30–4 mycelial growth. **(D)** Inhibition rate of *C. repens* extract on T30–4 mycelial growth. Different lowercase letters indicate statistically significant differences (P < 0.05). P values were calculated using one-way ANOVA. Each value represents the mean ± SD.

**Figure 1** illustrates the effects of extracts from 11 wild plant species on the mycelial growth of *Phytophthora infestans* strain T30–4 at a concentration of 1 mg/mL. The results demonstrate that all 11 plant extracts inhibited the mycelial growth of T30–4 to varying degrees (**Figure 1A**). The inhibition rates, ranked from highest to lowest, were as follows: *Artemisia lavandulaefolia* (88.3%) > *Clinopodium repens* (81.3%) > *Senecio scandens* (48.0%) > *Debregeasia orientalis* (46.6%) > *Synedrella nodiflora* (46.5%) > *Aster baccharoides* (45.3%) > *Galinsoga quadriradiata* (44.0%) > *Eschenbachia japonica* (39.5%) > *Sonchus oleraceus* (34.7%) > *Ageratina adenophora* (33.3%) > *Laggera pterodonta* (30.2%) (**Figure 1B**). The inhibition rate of the solvent negative control group was 4.1%, which was negligible. **Figure 2** presents the effects of different concentrations of extracts from *A. lavandulaefolia* and *C. repens* on the mycelial growth of T30-4. The results indicate that both extracts exhibited notable anti-oomycete activity against T30–4 across various concentrations (**Figures 2A, B**). At concentrations of 2.0 mg/mL, 1.0 mg/mL, and 0.5 mg/mL, the inhibition rates of *A. lavandulaefolia* extract against T30–4 were 91.9%, 88.3%, and 80.5%, respectively, while those of *C. repens* extract were 85.6%, 81.3%, and 72.3%, respectively. The inhibitory activity of both extracts showed a positive correlation with concentration (**Figures 2C, D**).

### 3.2 Anti-oomycete activity of different polarity fractions from *A. lavandulaefolia* and *C. repens*

**Figure 3:**
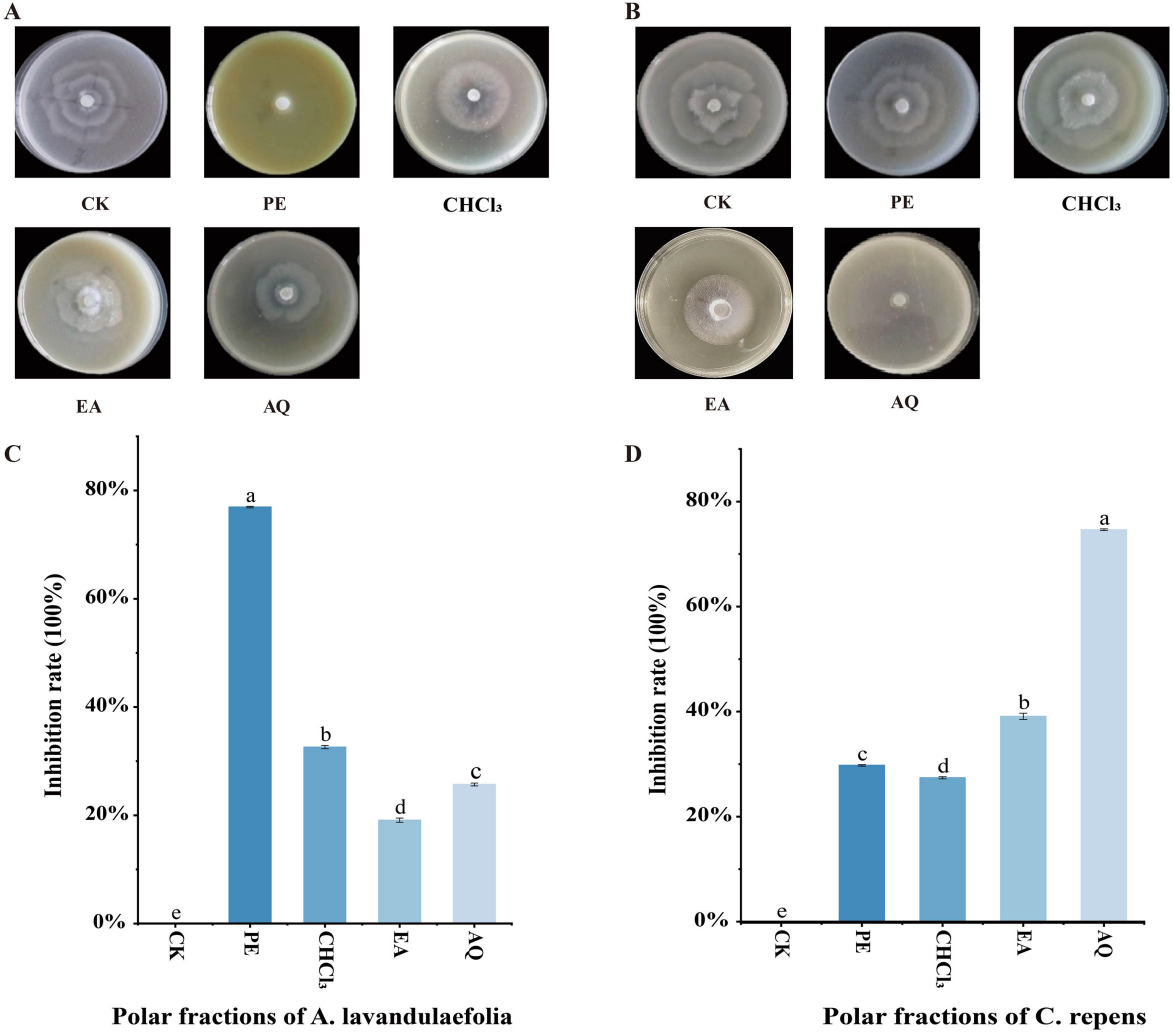
Inhibition of T30–4 mycelial growth by four polar fractions of the extracts from *A. lavandulaefolia* and *C. repens* at a concentration of 1 mg/mL. **(A)** Mycelial growth of T30–4 treated with four polar fractions of the *A. lavandulaefolia* extract. **(B)** Mycelial growth of T30–4 treated with four polar fractions of the *C. repens* extract. **(C)** Inhibition rates of the four polar fractions of the *A. lavandulaefolia* extract against T30–4 mycelial growth. **(D)** Inhibition rates of the four polar fractions of the *C. repens* extract against T30–4 mycelial growth. Different lowercase letters indicate significant differences (P < 0.05). P-values were calculated using one-way ANOVA. Each value represents the mean ± SD.

**Figure 3** illustrates the effects of the four polarity fractions from the extracts of *A. lavandulaefolia* and *C. repens* on the mycelial growth of *P. infestans* strain T30–4 at a concentration of 1 mg/mL. The results showed that all four polarity fractions from both plant extracts inhibited the mycelial growth of T30–4 to varying degrees (**Figures 3A, B**). For *A. lavandulaefolia*, the order of anti-oomycete activity from highest to lowest was the petroleum ether fraction (PE), chloroform fraction (CHCl_3_), aqueous fraction (AQ), and ethyl acetate fraction (EA), with corresponding inhibition rates of 76.9%, 32.6%, 25.7%, and 19.1%, respectively (**Figure 3C**). In contrast, for *C. repens*, the order was the AQ, EA, PE, and CHCl_3_, with inhibition rates of 74.6%, 39.1%, 29.7%, and 27.4%, respectively (**Figure 3D**). These findings indicate that the components inhibitory against T30–4 in the *A. lavandulaefolia* extract are primarily present in the petroleum ether fraction, whereas the active components in the *C. repens* extract are mainly concentrated in the aqueous fraction.

### 3.3 Determination of IC_50_ values of the petroleum ether fraction of *A. lavandulaefolia* and the aqueous fraction of *C. repens* against *P. infestans* isolate T30-4

**Figure 4:**
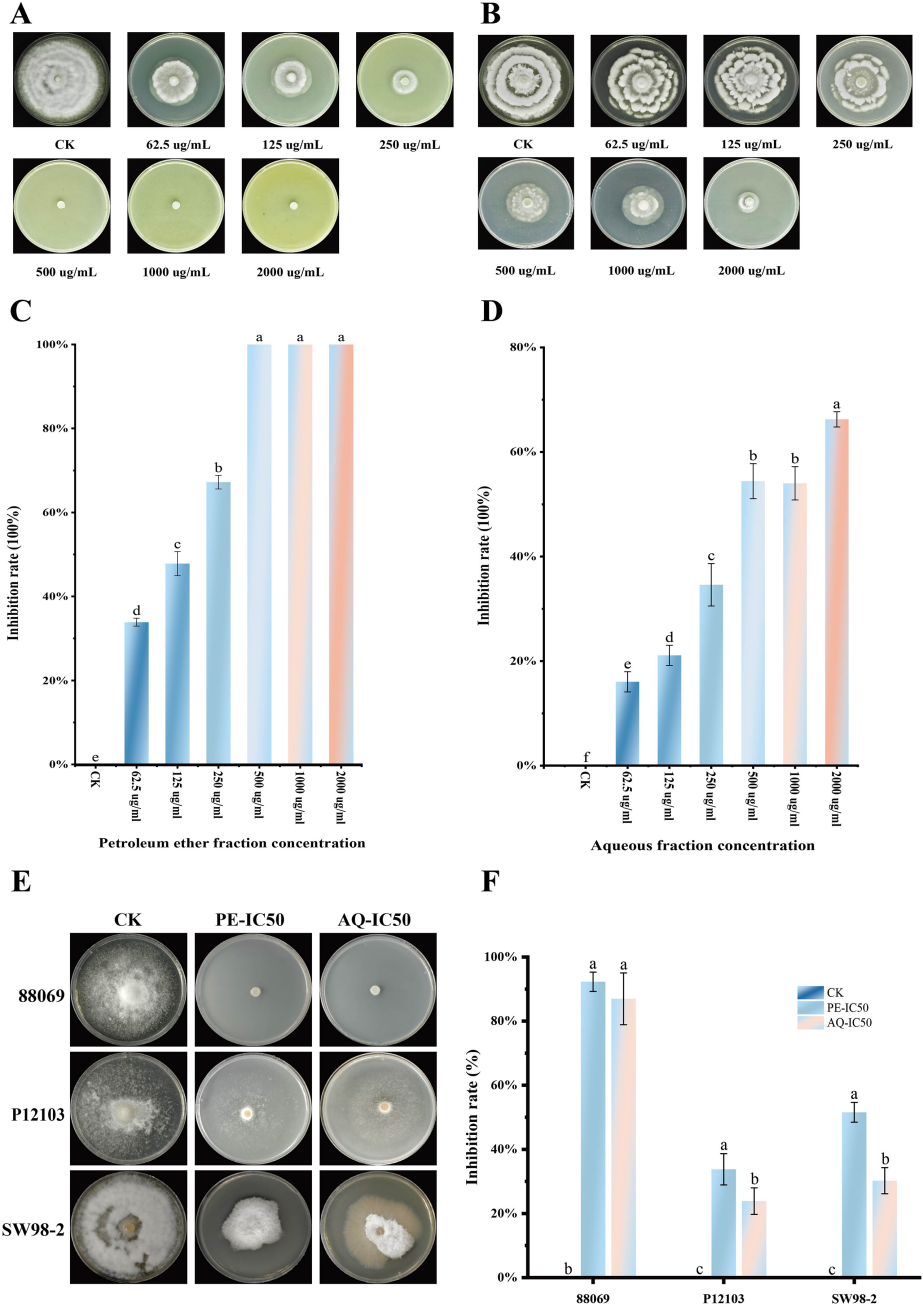
Inhibition of mycelial growth of *P. infestans* by petroleum ether fraction of *A. lavandulaefolia* and aqueous fraction of *C. repens* at different concentrations. **(A)** Mycelial growth of strain T30–4 treated with different concentrations of the PE from *A. lavandulaefolia*. **(B)** Mycelial growth of strain T30–4 treated with different concentrations of the AQ from *C. repens*. **(C)** Inhibition rate of the PE from *A. lavandulaefolia* on mycelial growth of strain T30-4. **(D)** Inhibition rate of the AQ from *C. repens* on mycelial growth of strain T30-4. **(E)** Mycelial growth of strain 88069, P12103, SW98–2 treated with IC_50_ values of the PE from *A. lavandulaefolia* and the AQ from *C. repens*. **(F)** Inhibition rate of the IC_50_ values of the PE from *A. lavandulaefolia* and the AQ from *C. repens* on mycelial growth of strain 88069, P12103, SW98-2. Different lowercase letters indicate significant differences (P < 0.05). P values were calculated using one-way ANOVA. Each value represents the mean ± SD.

The PE of *A. lavandulaefolia* and the AQ of *C. repens* significantly inhibited the mycelial growth of *P. infestans* isolate T30-4 (**Figures 4A, B**). When tested at concentrations of 2000, 1000, 500, 250, 125, and 62.5 µg/mL, the PE of *A. lavandulaefolia* exhibited inhibition rates of 100%, 100%, 100%, 67.21%, 47.81%, and 33.88%, respectively. Under the same concentration gradient, the AQ of *C. repens* showed inhibition rates of 66.24%, 54.01%, 54.43%, 34.60%, 21.10%, and 16.03%, respectively. A clear dose-dependent effect was observed, with colony diameters decreasing and inhibition rates increasing concomitantly with higher extract concentrations (**Figures 4C, D**). Statistical analysis using SPSS determined the IC_50_ values to be 115.78 µg/mL for the *A. lavandulaefolia* PE and 649.59 µg/mL for the *C. repens* AQ. Furthermore, at their respective IC_50_ concentrations, the *A. lavandulaefolia* PE (115.78 µg/mL) demonstrated inhibitory effects against additional *P. infestans* isolates, namely 88069, P12103, and SW98-2, with inhibition rates of 92.27%, 33.78%, and 51.26%, respectively (**Figure 4E**). Similarly, the *C. repens* AQ at its IC_50_ concentration (649.59 µg/mL) inhibited isolates 88069, P12103, and SW98-2, resulting in inhibition rates of 86.96%, 23.87%, and 30.22%, respectively (**Figure 4F**).

### 3.4 Effects of petroleum ether fraction from *A. lavandulaefolia* and aqueous fraction from *C. repens* on the mycelial morphology of T30-4

**Figure 5:**
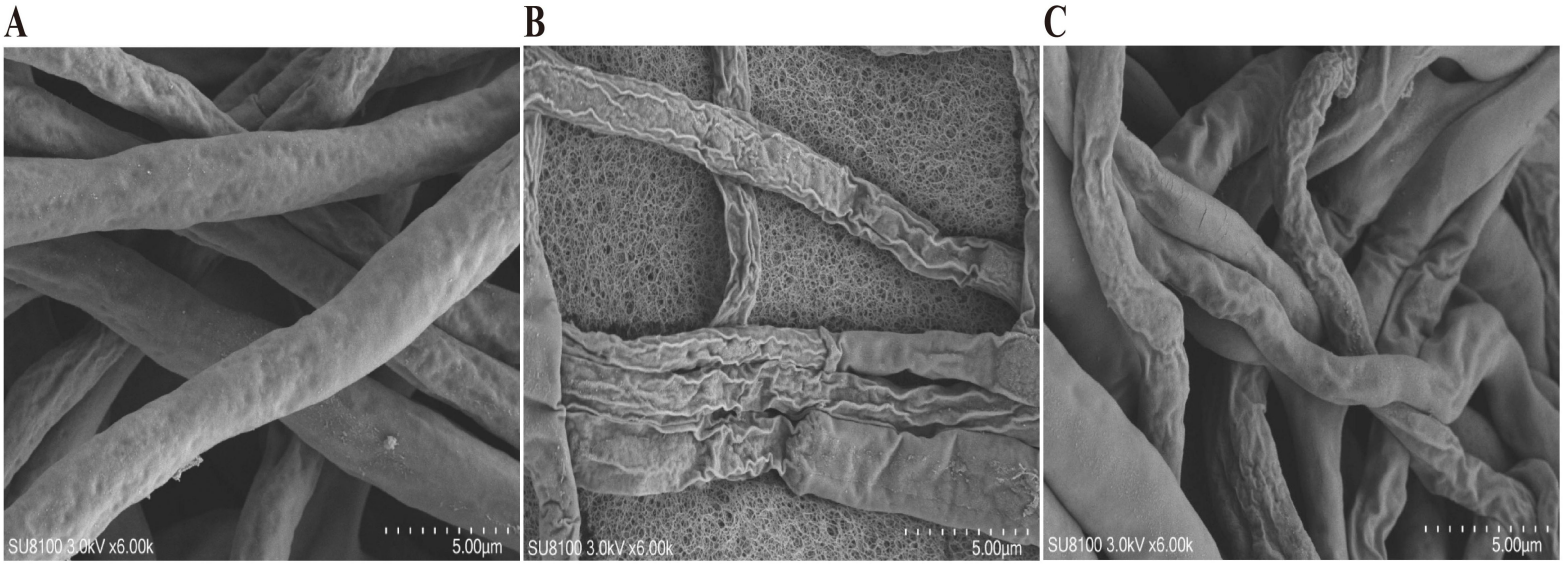
Scanning electron microscopy images of T30–4 treated with thePE of *A. lavandulaefolia* and the AQ of *C. repens*. **(A)** Blank control. **(B)** Treatment with the PE of *A. lavandulaefolia*. **(C)** Treatment with the AQ of *C. repens*.

Scanning electron microscopy (SEM) observations revealed that after 7 days of treatment with the PE of *A. lavandulaefolia* and the AQ of *C. repens*, the mycelial morphology of T30–4 underwent significant alterations. In the CK (**Figure 5A**), the hyphae exhibited a smooth surface, uniform thickness, and intact morphology. In contrast, hyphae treated with the IC_50_ concentrations of the PE of *A. lavandulaefolia* and the AQ of *C. repens* (**Figures 5B, C**) displayed an uneven thickness, a distorted and wrinkled appearance, and a rough surface. It is noteworthy that the impact on mycelial morphology was more pronounced for the PE of *A. lavandulaefolia*.

### 3.5 Effects of the petroleum ether fraction of *A. lavandulaefolia* and the aqueous fraction of *C. repens* on sporangia germination of T30-4

**Figure 6:**
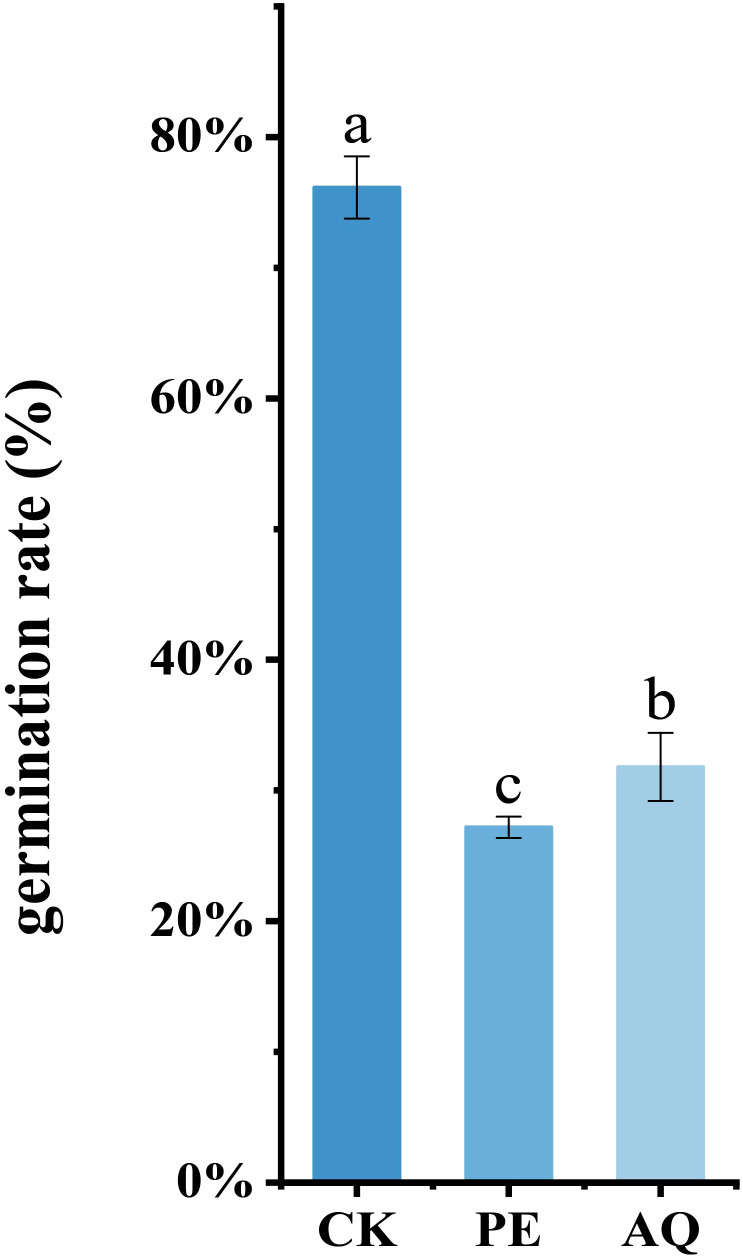
Spore germination rate of T30–4 treated with the PE of *A. lavandulaefolia* and AQ of *C. repens* at their respective IC_50_ concentrations. Different lowercase letters indicate statistically significant differences (P < 0.05) as determined by one-way ANOVA. Each value represents the mean ± SD.

The sporangia germination rate in the control group was 76.13%. After treatment with the PE of *A. lavandulaefolia* and the AQ of *C. repens* at their respective IC_50_ concentrations, the sporangia germination rates decreased significantly to 27.19% and 31.80%, respectively (**Figures 6A, B**). These results indicated that both the PE of *A. lavandulaefolia* and the AQ of *C. repens* can significantly inhibit the germination of *P. infestans* sporangia.

### 3.6 Effects of the petroleum ether fraction of *A. lavandulaefolia* and the aqueous fraction of *C. repens* on the pathogenicity of T30-4

**Figure 7:**
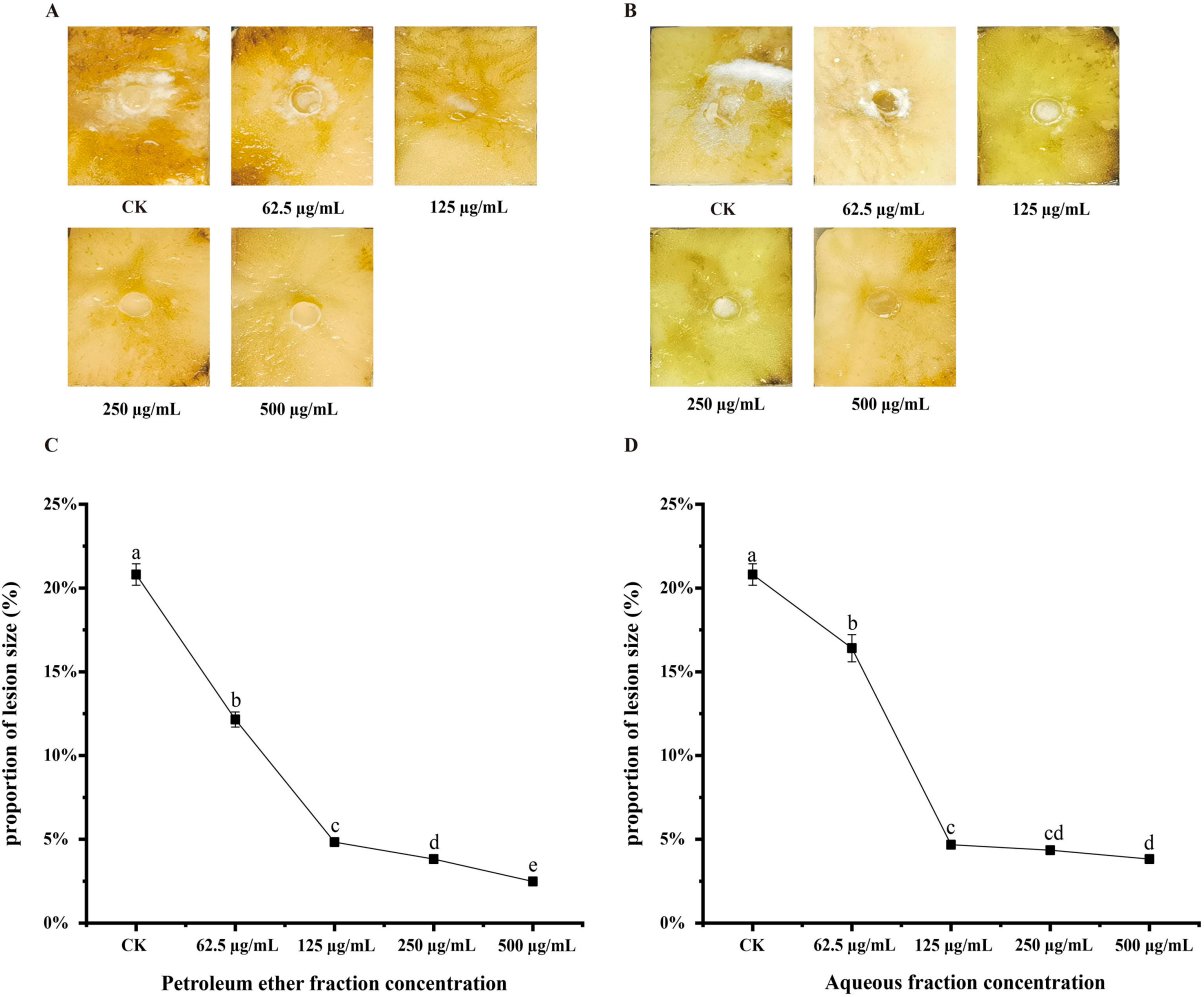
PE of *A. lavandulaefolia* and AQ of *C. repens* reduce the pathogenicity of T30-4. **(A)** Effect of different concentrations of PE from *A. lavandulaefolia* on potato tuber late blight. **(B)** Effect of different concentrations of AQ from *C. repens* on potato tuber late blight. **(C)** Lesion ratio of potato tubers infected with T30–4 after treatment with different concentrations of PE from *A. lavandulaefolia*. **(D)** Lesion ratio of potato tubers infected with T30–4 after treatment with different concentrations of AQ from *C. repens*. Different lowercase letters indicate significant differences (P < 0.05). P-values were calculated using one-way ANOVA. Each value represents the mean ± SD.

After treating potato tubers with different concentrations of the PE of *A. lavandulaefolia* and the AQ of *C. repens* for five days, varying degrees of infection were observed in the tubers (**Figure 7**). At extract concentrations of 500, 250, 125, and 62.5 μg/mL, the lesion area ratios of tubers treated with the PE of *A. lavandulaefolia* were 2.48%, 3.82%, 4.83%, and 12.15%, respectively (**Figure 7C**), while those treated with the AQ of *C. repens* showed lesion area ratios of 3.82%, 4.35%, 4.68%, and 16.41%, respectively (**Figure 7D**). With increasing extract concentrations, the lesion size, mycelial layer thickness, and degree of tuber rot decreased, and were significantly lower than those in the CK group (20.81%, P < 0.05). Thus, treatment of T30–4 with the PE of *A. lavandulaefolia* and the AQ of *C. repens* reduced the pathogenicity of T30-4.

### 3.7 Stress analysis of T30–4 treated with petroleum ether fraction of *A. lavandulaefolia* and aqueous fraction of *C. repens*

**Figure 8:**
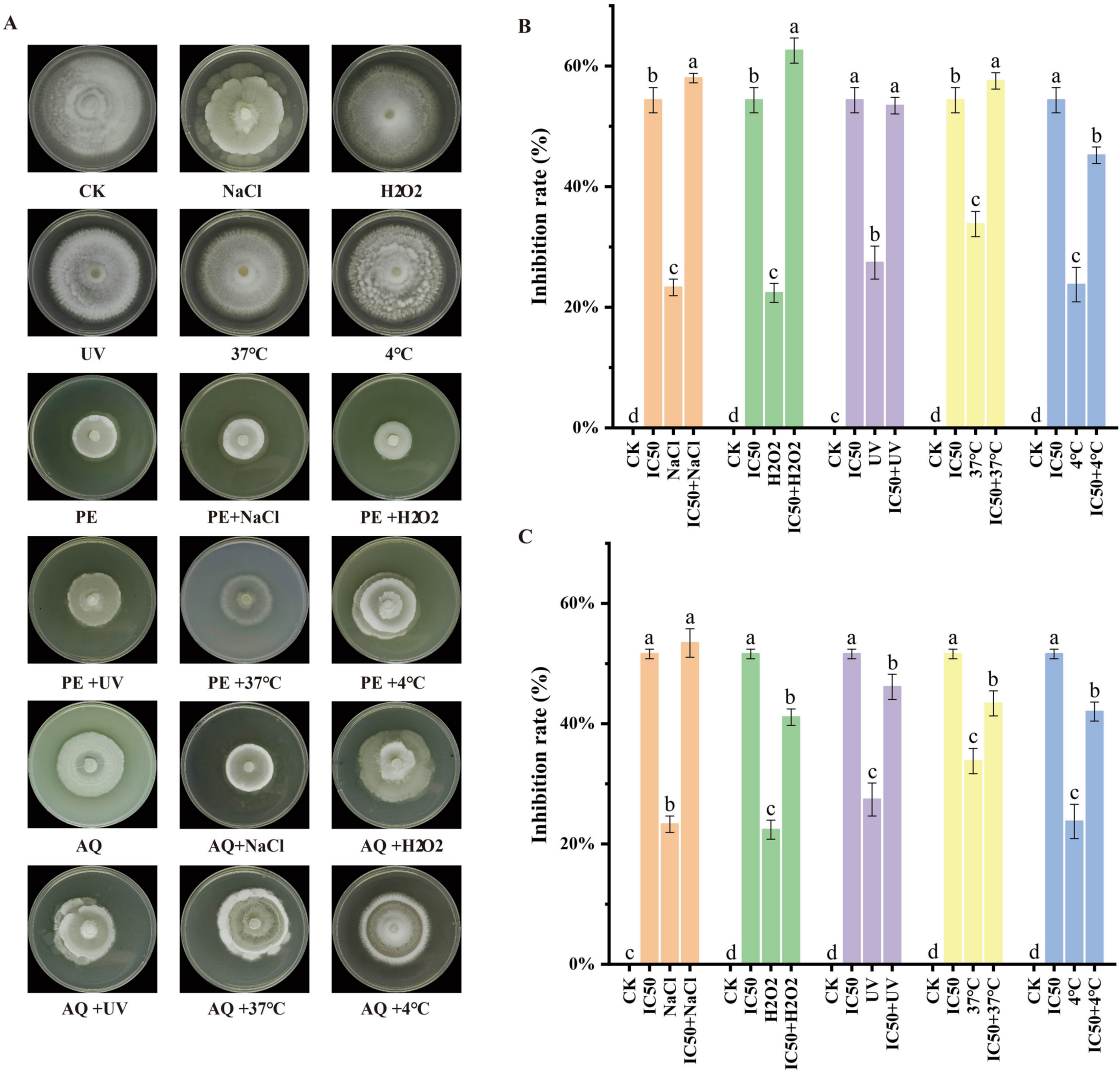
The PE of *A. lavandulaefolia* and the AQ of *C. repens* enhance the sensitivity of T30–4 to stress. **(A)** Mycelial growth of T30–4 under different stress treatments and co-treatments with the IC_50_ of the PE of *A. lavandulaefolia* or the AQ of *C. repens*. **(B)** Inhibition rate of mycelial growth of T30–4 by co-treatment of the IC_50_ of the PE of *A. lavandulaefolia* with different stresses. **(C)** Inhibition rate of mycelial growth of T30–4 by co-treatment of the IC_50_ of the AQ of *C. repens* with different stresses. Different lowercase letters indicate significant differences (P < 0.05) as calculated by one-way ANOVA. Each value represents the mean ± SD.

Under various stress conditions (UV, NaCl, H_2_O_2_, low temperature, and high temperature), the growth rate of T30–4 was significantly reduced compared to the CK group (**Figure 8A**). These results indicate that the pathogen is sensitive to these stress conditions. Subsequently, treatment with the PE of *A. lavandulaefolia* and the AQ of *C. repens* at IC_50_ concentrations was applied. It was observed that these fractions markedly enhanced the sensitivity of T30–4 to adverse conditions such as UV, NaCl, H_2_O_2_, low temperature, and high temperature, leading to a significant slowdown in its growth rate (**Figures 8B, C**). These findings suggest that the PE of *A. lavandulaefolia* and the AQ of *C. repens* may exhibit promising anti-oomycete efficacy against T30–4 under specific unfavorable conditions at their respective IC_50_ concentrations.

### 3.8 Combined antibacterial effects of the petroleum ether fraction of *A. lavandulaefolia* and the aqueous fraction of *C. repens* with infinito against T30-4

**Figure 9:**
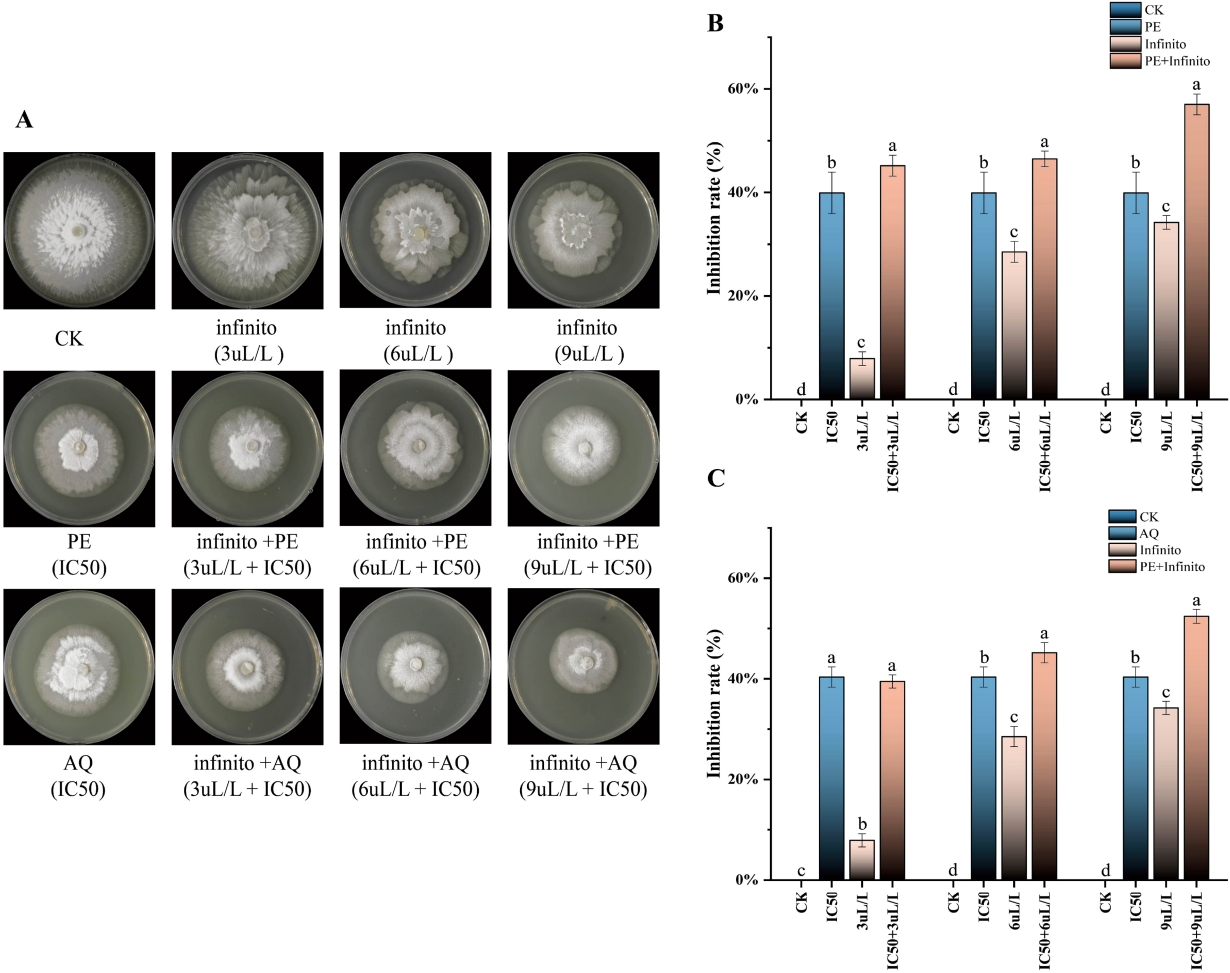
Combined effects of the petroleum ether fraction of *A. lavandulaefolia* and the aqueous fraction of *C. repens* with Infinito against strain T30-4. **(A)** Mycelial growth of T30–4 treated with different concentrations of Infinito alone or in combination with the IC_50_ of the petroleum ether fraction of *A. lavandulaefolia* or the aqueous fraction of *C. repens*. **(B)** Inhibition rate of mycelial growth of T30–4 treated with different concentrations of Infinito alone or in combination with the IC_50_ of the petroleum ether fraction of *A. lavandulaefolia*. **(C)** Inhibition rate of mycelial growth of T30–4 treated with different concentrations of Infinito alone or in combination with the IC_50_ of the aqueous fraction of *C. repens*. Different lowercase letters indicate significant differences (P < 0.05). P values were calculated using one-way ANOVA. Each value represents the mean ± SD.

Infinito was selected for this study due to its widespread application in controlling potato late blight in recent years. Infinito exhibited good inhibitory activity against *P. infestans*, and its inhibition rate changed in a dose-dependent manner with increasing Infinito concentration (**Figure 9A**). The combination of Infinito with the petroleum ether fraction of *A. lavandulaefolia* (at its IC_50_) showed a favorable inhibitory effect (**Figure 9B**). When the concentrations were 3, 6, and 9 μL/L, the Q values were 1.0118, 0.8150, and 0.9403, respectively. The interaction between Infinito and the petroleum ether fraction of *A. lavandulaefolia* was additive (0.85 < Q < 1.15), indicating that the petroleum ether fraction of *A. lavandulaefolia* can be used in combination with Infinito to reduce the dosage of the latter. Similarly, the combination of Infinito with the aqueous fraction of *C. repens* (at its IC_50_) also demonstrated a good inhibitory effect (**Figure 9C**). At concentrations of 3, 6, and 9 μL/L, the Q values were 0.8760, 0.8680, and 0.8626, respectively. The interaction between Infinito and the aqueous fraction of *C. repens* was additive (0.85 < Q < 1.15), suggesting that the aqueous fraction of *C. repens* can be combined with Infinito to lower its required application rate.

### 3.9 LC-MS analysis results of the petroleum ether fraction of *A. lavandulaefolia* and the aqueous fraction of *C. repens*

The chemical components of the PE of *A. lavandulaefolia* and the AQ of *C. repens* were analyzed using LC-MS technology. Compound identification was performed with the aid of Agilent MassHunter Qualitative Analysis software (version B.08.00). The acquired MS/MS spectra were matched against the Agilent Personal Compound Database and Library (PCDL) for natural products. The matching parameters were set as follows: precursor ion mass deviation ≤ 5 ppm, MS/MS fragment ion deviation ≤ 0.02 Da. Only compounds with a match score ≥ 80 were included in the putative identification. Based on the accurate relative molecular masses, retention times, and MS/MS fragmentation information provided by LC-MS, and combined with comparisons to relevant literature data, 20 compounds were tentatively identified from the petroleum ether fraction of *A. lavandulaefolia* (**Table 2**. LC-MS analysis of petroleum ether fraction of *Artemisia lavandulaefilia*). These are primarily small-polarity components, encompassing structural types such as flavonoids, phenolics, terpenoids, and long-chain fatty acids. Similarly, 20 compounds were tentatively identified from the aqueous fraction of *C. repens* (**Table 3**. LC-MS analysis of aqueous fraction of *Clinopodium repens*), which mainly consist of polar components, including organic acids, flavonoids, and their glycosides.

**Table 2:** LC-MS analysis of petroleum ether fraction of *Artemisia lavandulaefilia*.

No.	Name	RT (min)	Formula	M/Z	Addition Ions	Ionization mode	DB (ppm)
1	Maltose	2.7061	C_12_H_22_O_11_	341.287	[M-H]-	ESI-	0.21
2	D-(-)-Quinic acid	2.8161	C_7_H_12_O_6_	191.165	[M-H]-	ESI-	0.39
3	Cryptochlorogenic acid	5.4701	C_16_H_18_O_9_	353.307	[M-H]-	ESI-	0.64
4	Kaempferol-3-gentiobioside	5.8575	C_27_H_30_O_16_	611.523	[M+H]+	ESI+	-1.21
5	Vicenin-2	6.0834	C_27_H_30_O_15_	653.521	[M+AC-H]-1	ESI-	1.02
6	Myricitrin	6.1434	C_21_H_20_O_12_	465.368	[M+H]+	ESI+	0.79
7	Quercetin-7-O-β-D-glucopyranoside	6.1614	C_21_H_20_O_12_	463.354	[M-H]-	ESI-	0.68
8	Orientin	6.3714	C_21_H_20_O_11_	449.372	[M+H]+	ESI+	0.99
9	3,5-Di-O-caffeoylquinic acid	6.5944	C_25_H_24_O_12_	515.437	[M-H]-	ESI-	-1.26
10	Tiliroside	7.0866	C_30_H_26_O_13_	593.510	[M-H]-	ESI-	0.83
11	Vitexin2''-O-p-coumarate	7.1719	C_30_H_26_O_12_	577.493	[M-H]-	ESI-	0.45
12	Fraxinellone	7.2405	C_14_H_16_O_3_	231.264	[M-H]-	ESI-	0.33
13	Caffeic acid ethyl ester	8.2457	C_11_H_12_O_4_	207.209	[M-H]-	ESI-	0.48
14	Groenlandicine	9.9998	C_19_H_16_NO_4_	322.327	[M+H]+	ESI+	0.66
15	Vitexicarpin	10.5649	C_19_H_18_O_8_	373.338	[M-H]-	ESI-	-0.39
16	Parthenolide	12.4116	C_15_H_20_O_3_	249.317	[M+H]+	ESI+	0.57
17	11-hydroxy-sugiol	14.5664	C_20_H_28_O_3_	315.428	[M-H]-	ESI-	0.49
18	Ambroxane	16.2725	C_16_H_28_O	235.387	[M-H]-	ESI-	0.33
19	α-Linolenic acid	24.6867	C_18_H_30_O_2_	277.396	[M-H]-	ESI-	0.51
20	Linoleic acid	28.0411	C_18_H_32_O_2_	279.441	[M-H]-	ESI-	0.61

**Table 3:** LC-MS analysis of aqueous fraction of *Clinopodium repens*.

No.	Name	RT (min)	Formula	M/Z	Addition Ions	Ionization mode	DB (ppm)
1	5,7,3'-Trihydroxy-6,4',5'-trimethoxyflavone	18.0332	C_18_H_16_O_8_	359.311	[M-H]-	ESI-	0.33
2	Baicalin	17.1602	C_21_H_18_O_11_	447.358	[M+H]+	ESI+	0.08
3	Buddleoside	19.9765	C_28_H_32_O_14_	615.542	[M+H]+	ESI+	0.29
4	Caffeic acid	12.3093	C_9_H_8_O_4_	179.152	[M-H]-	ESI-	0.18
5	Citric acid	4.9103	C_6_H_8_O_7_	191.120	[M-H]-	ESI-	-0.31
6	Cryptochlorogenic acid	8.1381	C_16_H_18_O_9_	353.301	[M-H]-	ESI-	0.33
7	D-(-)-Quinic acid	3.599	C_7_H_12_O_6_	191.162	[M-H]-	ESI-	1.01
8	DL-Tartaric acid	3.7122	C_4_H_6_O_6_	149.080	[M-H]-	ESI-	0.86
9	Ethyl gallate	7.3387	C_9_H_10_O_5_	197.175	[M-H]-	ESI-	0.53
10	Galactaric Acid	3.4401	C_6_H_10_O_8_	209.135	[M-H]-	ESI-	0.29
11	Isorhamnetin-3-O-neohespeidoside	15.2225	C_28_H_32_O_16_	625.539	[M+H]+	ESI+	0.71
12	Lithospermic acid	14.7099	C_27_H_22_O_12_	537.451	[M-H]-	ESI-	0.56
13	L-Phenylalanine	7.0042	C_9_H_11_NO_2_	166.185	[M+H]+	ESI+	0.42
14	L-Ribose	3.4267	C_5_H_10_O_5_	149.132	[M-H]-	ESI-	0.13
15	Malic acid	3.9038	C_4_H_6_O_5_	133.084	[M-H]-	ESI-	1.09
16	Myricitrin	15.3184	C_21_H_20_O_12_	465.375	[M+H]+	ESI+	0.28
17	Orientin	17.6366	C_21_H_20_O_11_	449.371	[M+H]+	ESI+	0.57
18	Pinobanksin	17.6638	C_15_H_12_O_5_	273.249	[M+H]+	ESI+	0.37
19	Quercetin-7-O-β-D-glucopyranoside	15.2641	C_21_H_20_O_12_	463.371	[M-H]-	ESI-	0.07
20	Salvianolic acid A	18.0903	C_26_H_22_O_10_	493.44	[M-H]-	ESI-	0.19

## 4 Discussion

*Phytophthora infestans* (Mont) de Bary, the causal agent of potato late blight, is a heterothallic oomycete. Currently, three main types exist: A1, A2, and self-fertile (SF) types (Fry, 2008; Li, 2019). Prior to the 1940s, potato late blight was predominantly caused by the A1 mating type. It was not until 1956 that the presence of oospores was first confirmed in large quantities in central Mexico, demonstrating the existence of the A2 mating type (Gallegly and Niederhauser, 1959). The emergence of the A2 mating type signifies the potential for the development of strains or physiological races with enhanced virulence and variability, thereby accelerating pathogen evolution (Fry and Goodwin, 1997). Current control strategies for potato late blight primarily rely on cultivating resistant varieties and applying chemical pesticides. However, due to the rapid genetic variation of *P. infestans*, strains capable of overcoming all known resistance (R) genes have emerged (Liu et al., 2010). The isolate T30–4 used in this study is a typical representative of the US-1 lineage and A2 mating type, exhibiting virulence against the vast majority of R genes (Haas et al., 2009). SW98–2 is an A1 mating type isolate collected from Xichang, Sichuan, which also shows virulence against most R genes (Zhu, 2004). Isolates 88069 and P12103 are self-fertile types virulent against most R genes, with P12103 demonstrating stronger infectivity on tomato (Zhu et al., 2023). Long-term reliance on chemical control alone not only has limitations in managing potato late blight but also leads to environmental pollution and pesticide residue issues (Xia et al., 2022). Consequently, the search for green and safe bio-pesticides is imperative for controlling potato diseases. In this study, an activity-guided approach was employed to conduct preliminary screening of ethanol extracts from 11 wild plant species for anti-oomycete activity against *P. infestans*. The results revealed significant differences in their ability to inhibit mycelial growth, with the extracts from *Artemisia lavandulaefolia* and *Clinopodium repens* demonstrating significantly higher anti-oomycete activity compared to other plant extracts. This finding underscores the immense potential of plant biodiversity as a source of natural antifungal agents (Dixon, 2001). Based on the inhibition rates of the extracts, we selected *A. lavandulaefolia* and *C. repens* for further investigation. This strategy aligns with the research logic of efficiently discovering bioactive compounds by prioritizing the study of the most promising candidates (Hostettmann et al., 1986).

Subsequently, gradient extraction of *A. lavandulaefolia* and *C. repens* extracts was performed using solvents of increasing polarity (petroleum ether, chloroform, ethyl acetate) to enrich bioactive constituents (Hostettmann et al., 1986). Compared with other fractions, the petroleum ether fraction of *A. lavandulaefolia* extract and the aqueous fraction of *C. repens* extract exhibited superior anti-oomycete activity. This may be attributed to the enrichment of low- to medium-polarity compounds (such as phenolics, flavonoids, terpenoids, etc.) in the petroleum ether fraction of *A. lavandulaefolia*, and the enrichment of high-polarity compounds (such as phenolics, flavonoid glycosides, and terpenoids, etc.) in the aqueous fraction of *C. repens*, which have been widely reported to possess antimicrobial activity (Xiong, 2011; Chen et al., 2024). This purification step not only enhanced the efficacy but also simplified the complex mixture required for subsequent chemical analysis, thereby directly facilitating the identification of the genuine active components.

A multidimensional evaluation was conducted on the anti-oomycete activity of the petroleum ether fraction of *A. lavandulaefolia* extract and the aqueous fraction of *C. repens* extract against *P. infestans*, focusing on the inhibition of mycelial growth, sporangia germination, and pathogenicity. Scanning electron microscopy revealed that treatment with the IC_50_ of the petroleum ether fraction of *A. lavandulaefolia* extract and the aqueous fraction of *C. repens* extract caused significant morphological alterations in the hyphae of *P. infestans*. These morphological changes may be attributed to the disruption of the cellular structure of the hyphae by the extracts. This disruption could lead to the exosmosis of cellular contents, resulting in collapse and ultimately causing distortion and wrinkling of the cells (Shi et al., 2025). These findings provide novel insights into the cellular mechanisms by which the petroleum ether fraction of *A. lavandulaefolia* extract and the aqueous fraction of *C. repens* extract inhibit *P. infestans*. Sporangia are crucial structures for the production and containment of proliferative sporangia in the pathogen; therefore, assessing the impact of these extracts on sporangial activity is a powerful means to evaluate their anti-*Phytophthora* efficacy (Aylor et al., 2001). At the IC_50_ concentration, the sporangia germination rates were 27.19% and 31.80%, respectively, significantly lower than the CK rate of 76.13%. This result aligns with findings from Zhang (2021), who studied the inhibitory effect of ethylicin on potato late blight, and Zhu et al. (2023), who investigated the anti-oomycete activity of scopolamine against *P. infestans*, suggesting a possible association with the loss of cell membrane integrity. The potato cultivar ‘Qingshu 9’ was previously known for its high resistance to late blight, but reports indicate that this resistance is gradually diminishing. In this study, detached potato tubers were susceptible to *P.infestans* infection. The petroleum ether fraction of *A. lavandulaefolia* extract and the aqueous fraction of *C. repens* extract reduced the pathogenicity of *P. infestans* in potato tubers, which may also be attributed to the extracts damaging the hyphal cell structure, thereby affecting the normal physiological architecture and function of the pathogen. Stress analysis results demonstrated that both extract fractions exhibited favorable inhibitory effects against the pathogen, providing a basis for subsequent field trials (under various adverse conditions) involving the petroleum ether fraction of *A. lavandulaefolia* extract and the aqueous fraction of *C. repens* extract. Infinito is a chemical pesticide widely used in recent years to control potato late blight. Co-application studies showed an additive effect between the two polar fractions and Infinito (Wang L. et al., 2025); their combination could reduce the required dosage of the latter, although this needs validation through later field experiments. The inhibition of sporangia germination is particularly critical, as it represents the core link for the rapid spread and epidemic outbreak of late blight in the field (Zhang, 2021). The observed attenuation of *P. infestans* pathogenicity on potato tubers further confirms the practical value of these extracts, indicating that their mechanism of action extends beyond merely inhibiting *in vitro* growth to interfering with key infection processes. The above results provide comprehensive evidence for the potential of the petroleum ether fraction of *A. lavandulaefolia* extract and the aqueous fraction of *C. repens* extract as botanical protective agents, offering a reliable basis for subsequent field experiments.

He et al. (2021) investigated the antifungal activity of five *Artemisia* species against potato late blight and found that the petroleum ether fraction of *Artemisia dubia* crude extract exhibited strong inhibitory activity (100% inhibition) against *P. infestans* on two potato plants, Pn5502 and Pn7604. However, no further study was conducted on the active substances present in this fraction. Our study confirms the significant inhibitory effect of the PE of *A. lavandulaefolia* against *P. infestans*. LC-MS analysis of this fraction led to the tentative identification of 30 compounds based on molecular weight and fragment ion patterns, predominantly medium and low polarity compounds, including flavonoids, phenolics, terpenoids, and small molecular acids. Among them were known classes of antifungal compounds. For example, D-(-)-Quinic acid, isolated from *Cimicifuga dahurica*, has been reported to exhibit inhibitory activity against *Botrytis cinerea* and *Rhizoctonia solani* and plays an important role in the plant’s natural immune system against fungi by regulating the synthesis of other antimicrobial compounds (Chong et al., 2009). Cryptochlorogenic acid showed inhibitory activity against *Staphylococcus aureus* (Li et al., 2018). Caffeic acid derivatives (e.g., 3,5-Di-O-caffeoylquinic acid, Caffeic acid ethyl ester) have been reported to disrupt fungal cell membrane integrity, inhibit key enzymes, or induce plant defense responses (Ojeda-Contreras et al., 2008; Lee et al., 2013; Burke et al., 1995; Johnson et al., 2004; Murtaza et al., 2014; Meyuhas et al., 2015; Velazquez et al., 2007; Alfarrayeh, 2021).

The aqueous fraction of the extract from *C. repens* was subjected to LC- MS analysis, leading to the preliminary identification of 20 compounds based on molecular weights and fragmentation patterns. Most of these were highly polar compounds, including flavonoids, phenolics, and terpenoids—classes known to possess certain antimicrobial and anti-inflammatory activities (Li et al., 2014). For instance, orientin isolated from *Desmodium heterocarpon* var. *stigosum* exerts its antifungal activity by inserting into the fungal cell membrane via hydrophobic interactions, disrupting its integrity and causing leakage of cellular contents (Le et al., 2023). Myricitrin has been identified as a key biomarker for inhibiting *Phytophthora ramorum* (Conrad et al., 2017). Quercetin-7-*O*-β-D-glucopyranoside can exhibit antifungal activity through multiple pathways such as interaction with cell membranes, inhibition of enzymatic activity, and disruption of microbial metabolism. Its activity spectrum covers several important plant pathogenic fungi (e.g., *Fusarium*, *Rhizoctonia*) and post-harvest pathogens (e.g., *Penicillium*) (Cushnie and Lamb, 2005; Kanwal et al., 2010; Hernández et al., 2021). The novelty of this study lies in revealing the potent anti-*P. infestans* activity of components derived from *A. lavandulaefolia* and *C. repens*, which are previously underexplored plant sources. The possible synergistic effects among the multiple compounds within this fraction may explain its high efficacy—a recognized advantage of botanical extracts over single-site synthetic fungicides—and could contribute to delaying the development of resistance in pathogen populations (Raveau et al., 2020; Su et al., 2023).

This study has certain limitations. Although the preliminarily identified compounds provide important clues for elucidating the antifungal active constituents of the petroleum ether fraction of *A. lavandulaefolia* and the aqueous fraction of *C. repens*, the tentative annotations based on LC-MS still have limitations in structural confirmation, as they cannot completely exclude the interference of isomers nor accurately determine the absolute configurations of the compounds (Li et al., 2025). Therefore, subsequent research should employ techniques such as nuclear magnetic resonance spectroscopy, high-resolution mass spectrometry, and circular dichroism spectroscopy to perform precise structural identification of the key active components. Based on this, systematic structure-activity relationship studies should be conducted to clarify the intrinsic correlation between active groups and antifungal activity (Yang et al., 2025; Xu et al., 2024). Furthermore, by systematically analyzing the transcriptome, metabolome, and epigenome of *Phytophthora infestans* before and after treatment with the extracts, the molecular regulatory network underlying the response of *P. infestans* to the extracts from *A. lavandulaefolia* and *C. repens* should be constructed. This will clarify the mechanism of action of the candidate compounds, as well as identify potential resistance-related genes and their evolutionary pathways. Ultimately, these findings will provide a solid theoretical basis for optimizing extract combination strategies, rationally designing fungicide rotation schemes, and effectively delaying the development of resistance (Quan et al., 2025; Lodi et al., 2025).

## 5 Conclusion

In this study, the ethanol extracts of 11 wild plants were screened via bioassay-guided fractionation, revealing that *Artemisia lavandulaefolia* and *Clinopodium repens* exhibited the strongest inhibitory effects on the mycelial growth of *Phytophthora infestans*. Subsequently, gradient extraction and anti-oomycete assays using different solvents were conducted, showing that the petroleum ether fraction of *A. lavandulaefolia* and the aqueous fraction of *C. repens* possessed the most potent anti-oomycete activity, highlighting their significant potential as novel botanical pesticides for controlling potato late blight. Moreover, the mechanisms of action were investigated from multiple dimensions, including mycelial morphology, spore germination rate, pathogenicity, stress response analysis, combination experiments with Infinito, and chemical composition analysis. The results demonstrated that these extracts function by disrupting the cellular structure of the pathogen, inhibiting spore dissemination, and reducing its pathogenic capability. They also maintained stable efficacy under various stress conditions, and their rich array of bioactive compounds likely contributes to efficient and resistance-resistant anti-oomycete effects through multi-target synergistic actions. This study provides a theoretical foundation for developing efficient and low-toxicity botanical pesticides against potato late blight. Future research should focus on the isolation and identification of core active monomers, structure-activity relationships, field efficacy evaluation, and in-depth investigation of the modes of action against the pathogen.

## Data Availability

The original contributions presented in the study are included in the article/supplementary material. Further inquiries can be directed to the corresponding author.
